# Editorial: The Superior Colliculus/Tectum: Cell Types, Circuits, Computations, Behaviors

**DOI:** 10.3389/fncir.2019.00039

**Published:** 2019-06-03

**Authors:** Karl Farrow, Tadashi Isa, Harald Luksch, Keisuke Yonehara

**Affiliations:** ^1^Neuro-Electronics Research Flanders, Leuven, Belgium; ^2^Department of Biology, Leuven Brain Institute, KU Leuven, Leuven, Belgium; ^3^VIB, Leuven, Belgium; ^4^imec, Leuven, Belgium; ^5^Department Neuroscience, Graduate School of Medicine, Kyoto University, Kyoto, Japan; ^6^Lehrstuhl für Zoologie, Technische Universität München, Freising, Germany; ^7^Department of Biomedicine, Aarhus University, Aarhus, Denmark; ^8^Danish Research Institute of Translational Neuroscience, Aarhus, Denmark

**Keywords:** superior colliculus, optic tectum, cell types, neuronal circuit, visual processing

The superior colliculus is an important brain structure that transforms sensory inputs into motor outputs for the purpose of directing orienting behaviors and attention. One special feature of the superior colliculus is that sensory and motor maps are aligned topologically across the two-dimensional layers, which facilitates the triggering of behavioral responses in coordination with the sensory environment. However, basic descriptions of physiological and anatomical properties of neurons in the superior colliculus, as well as the input-output relationship with other brain areas is still not satisfactory. Furthermore, the degree to which neuronal and circuitry properties are conserved across species or specialized for specific behaviors remains unclear.

The aim of this research topic is to highlight the breadth of research currently being performed on the superior colliculus across different animal species in the form of original articles, reviews, and perspectives. The research topic successfully collected 11 articles contributed by 48 authors. Among the collected papers in this research topic, two were performed in zebrafish, one in rats, three in mice, and five were about non-human primates.

First, although the superior colliculus (optic tectum in zebrafish) is often studied by testing how to respond to sensory stimuli or generate motor commands, the spontaneous activity of the neurons can provide insights into the spatial organization and functional connectivity within the superior colliculus (Marachlian et al.). Marachlian et al. provide a review of the zebrafish literature focusing on studies using large scale recordings of the spontaneous activity of neurons in the larval zebrafish optic tectum. This review highlights the unique advantages of the larval zebrafish as a model organism to monitor activity in the entire optic tectum simultaneously.

A clear advantage of using the superior colliculus to understand how retinal inputs are further processed is that neurons in the superficial layers of the superior colliculus receive this information directly, and it is localized relatively superficial of the brain, allowing for good experimental access. One key to addressing such research questions is the ability to label cell types for manipulating and dissecting neuronal pathways mediating individual information streams (Oliveira and Yonehara). Along these lines Villalobos et al. present a study investigating whether parvalbumin-positive neurons are a unique cell type within the mouse superior colliculus. They found that the mouse superior colliculus, parvalbumin-positive neurons are heterogeneous, comprising both GABAergic inhibitory and glutamatergic excitatory neurons, which is dissimilar to some other brain areas such as the cortex, hippocampus and striatum, where parvalbumin-positive neurons are almost exclusively inhibitory (Villalobos et al.).

Two of the most ubiquitous response features studied in the visual system, from the retina to visual cortex, are orientation and direction selectivity. In mice, recent studies have highlighted the organization and origin of these response features in the superior colliculus. Here Shi et al. demonstrated that the direction and orientation selectivity of collicular neurons increases during the transformation from membrane potential to spiking responses. Intriguingly, the degree of increase depends on the receptive field size of the neuron (Shi et al.). In macaque monkeys, Chen and Hafed detail how neurons in the superficial layers of the superior colliculus are tuned to orientation, contrast, and temporal flicker fusion characteristics. These findings suggest that the primate superior colliculus might mediate coarse, but rapid, object detection and identification that can either facilitate or inhibit orienting responses of the animal (Chen and Hafed).

In addition to the retina, the superior colliculus receives an abundance of inputs from a variety of brain areas likely for the purpose of modulating the triggering of behaviors in ethologically relevant ways. Here Heap et al. demonstrate that in zebrafish hypothalamic neurons provide inhibitory projects to optic tectum, and this pathway appears to gate predatory behavior of the fish. In addition, Matsumoto et al. reviewed the causal role of inputs from frontal eye field to superior colliculus of monkeys during saccade generation.

Reflecting the diverse non-retinal inputs from other brain areas, the activity of superior colliculus neurons can be affected by internal states. Here two studies in monkeys illustrate how learning and expected reward values affect neuronal activity in the superior colliculus. Griggs et al. demonstrate that neurons in the superior colliculus can discriminate objects based on reward values, and such modulation is likely provided by the basal ganglia. In addition, Sadeh et al. show that the spatial coding of visual and motor responses of neurons in the superior colliculus are altered by the introduction of a trained delay during a memory-delay saccade task.

Like its inputs the output pathways of the superior colliculus show remarkable diversity. In rats, Kaneshige et al. show that the superior colliculus provides direct input to the contralateral facial nucleus, and indirectly to central pattern generators that are involved in modulating vibrissal movements. In macaque monkeys, Elorette et al. provide compelling anatomical evidence that the superior colliculus projects via pulvinar nuclei to the lateral amygdala, providing a short route for salient visual information to influence emotions.

As illustrated by the topics and methods of the collected papers, the study of the superior colliculus involves diverse questions, research methods, and animal models, with each providing a unique prospective on its role. Our hope is that this research topic illuminates the current trends and future directions of this field. In particular, we hope this collection will inspire researchers to integrate knowledge gained from different species and approaches to inform the next steps to be taken, and questions to be asked in the coming years.

## Author Contributions

All authors listed have made a substantial, direct and intellectual contribution to the work, and approved it for publication.

### Conflict of Interest Statement

The authors declare that the research was conducted in the absence of any commercial or financial relationships that could be construed as a potential conflict of interest.

